# An empirical study of the *per capita* yield of science Nobel prizes: is the US era coming to an end?

**DOI:** 10.1098/rsos.180167

**Published:** 2018-05-09

**Authors:** Claudius Gros

**Affiliations:** Institute for Theoretical Physics, Goethe University Frankfurt, Frankfurt a.M., Germany

**Keywords:** Nobel prizes, predictive modelling, science of sciences

## Abstract

We point out that the Nobel prize production of the USA, the UK, Germany and France has been in numbers that are large enough to allow for a reliable analysis of the long-term historical developments. Nobel prizes are often split, such that up to three awardees receive a corresponding fractional prize. The historical trends for the fractional number of Nobelists per population are surprisingly robust, indicating in particular that the maximum Nobel productivity peaked in the 1970s for the USA and around 1900 for both France and Germany. The yearly success rates of these three countries are to date of the order of 0.2–0.3 physics, chemistry and medicine laureates per 100 million inhabitants, with the US value being a factor of 2.4 down from the maximum attained in the 1970s. The UK in contrast managed to retain during most of the last century a rate of 0.9–1.0 science Nobel prizes per year and per 100 million inhabitants. For the USA, one finds that the entire history of science Noble prizes is described on a *per capita* basis to an astonishing accuracy by a single large productivity boost decaying at a continuously accelerating rate since its peak in 1972.

## 1. Introduction

The ‘science of sciences’ has emerged over the last decades as a vibrant research field [1], in particular because it may open a route to ‘predict’ discoveries in research fields that have achieved a certain degree of maturity [2]. On an individual level, the investigations are focused mainly on measures quantifying the scientific excellence of a given scientist [3,4], as well as the future impact of a research publication [5,6]. It remains, however, a challenge to predict at which stage of her or his career a scientist will be likely to publish a breakthrough paper, if ever [7–9].

The situation changes when it comes to the overall performance of a country and its scientific institutions [1], which is determined not by the achievements of individuals, but by aggregate and hence averaged variables. The same holds for the history of Nobel prizes in the natural sciences for larger countries.^1^ Nobel prizes have been acquired by the USA, the UK, Germany and France at rates that are steady enough, as we will show here, that the time evolution of the respective Nobel prize productivity can be both modelled accurately and forecast for the years to come. Such an analysis allows for an improved understanding of the history of scientific discoveries. It also provides science managers insights on how the aggregate productivity of the scientific institutions of a country is developing.

One can model empirical data either via a straightforward fit or by approximating it by an underlying model [10]. An example of the first approach is the linear increase in record human life expectancy that has been observed to hold for more than a century [11]. It is, however, difficult to gauge how long a trend found empirically will continue to persist. The historical rise of human life expectancy, to come back to this example, has been predicted repeatedly to level off [11], yet it keeps going [12]. The underlying drivers in terms of continuing progress in the medical sciences seem to remain operative.

Here, we propose that the history of science Nobel prize success can be analysed by a sociophysical model describing two fundamental drivers, an underlying long-term productivity rate and an extended but otherwise temporary burst in science productivity. We believe that the trends resulting from this model for the next decade are relatively robust. Our results, in particular that the US *per capita* science productivity in terms of Nobel prizes in the natural sciences is continuing to decline, warrant a critical discussion.

## 2. Results

**Figure 1.**
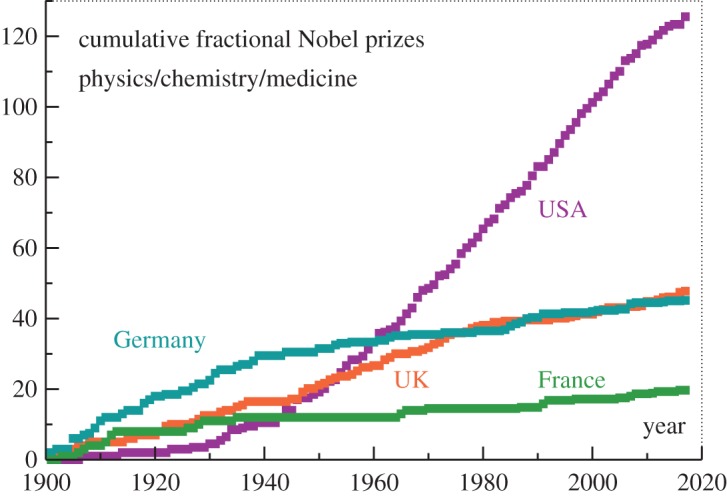
The cumulative number of physics, chemistry and medicine Nobel prizes per country. Prizes are attributed to the respective country according to the nationality of the recipients at the time of the announcement, with prizes obtained by more than one recipient accordingly divided. Note that the US population increased from 76 to 327 million during 1901–2017.

Nobel prizes are awarded as fractional prizes to up to three scientists. In figure 1, we have plotted the cumulative number of prizes, where the number of fractional laureates received in a given year was determined according to the nationality of the recipients at the time of the prize announcement (modulo multiple citizenships, when present). The USA has been leading, unsurprisingly, ever since the 1960s. To determine the relative contributions of the population size and of the excellence of the scientific institutions with regard to Nobel productivity, one needs to factor out the population size. This is particularly important when the population has been varying substantially. For the USA, to give an example, the population increased from 76 million in 1901, the year the Nobel prize was first awarded, to the present day value of 327 million.

**Figure 2.**
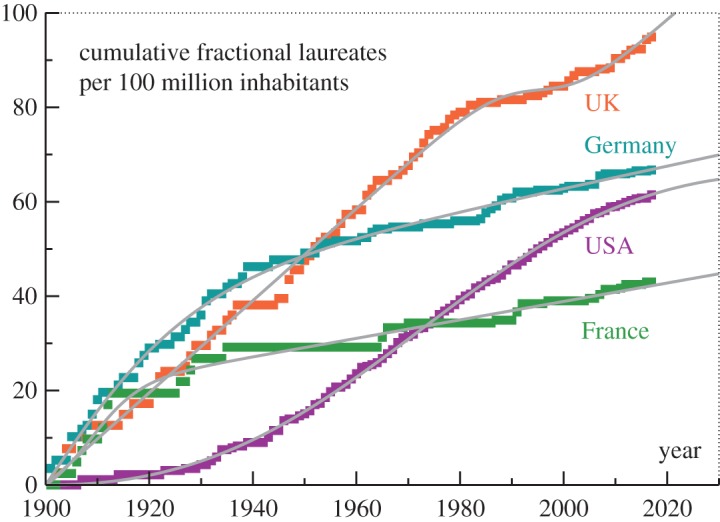
Science Nobel prizes per 100 million inhabitants. The historical population data at the time of the announcement were used and the obtained yearly increments were cumulatively added. The data can be modelled (grey lines; see equation (2.1)) by a superposition of a linear growth term and a one-time period of either increased (as for the USA, Germany and France) or reduced productivity (as for the UK), centred, respectively, around 1898, 1909, 1972 and 1995 for Germany, France, the USA and the UK.

For the yearly *per capita* yield we divided the fractional number of Nobel prizes a country received in a given year by the population the country of nationality had in the same year, interpolating whenever necessary the respective census data.^2,3,4,5^ The yearly counts obtained in this manner were added subsequently to a cumulative measure that has the advantage of fluctuating substantially less than the yearly increments. The resulting data are presented in figure 2, where we used a reference population of 100 million inhabitants. On a *per capita* basis the winner is the UK, with Germany coming second and the USA a close third. Prolonged periods without Nobel prizes show up in plateaus.

### 2.1. Modelling

The historical evolution of the *per capita* yield of fractional Nobel laureates presented in figure 2 can be fitted by a model,

2.1 $$\gamma t+\alpha g\left(\frac{t-t_{\max}}{\tau_{\max}}\right)-c_{0},$$

that incorporates a background of constant success ∝*γ*. The second term in (2.1) is proportional to the S-shaped logistic function *g*, with its derivative,

2.2 $$\frac{dg}{dx}=g(1-g),\;g(x)=\frac{1}{1+\exp(-x)},$$

corresponding either to a burst in productivity centred around $t_{\max}$, if *α*>0, or to a period with decreased Nobel success when *α*<0. The respective decay time is $\tau_{\max}$. The constant *c*_0_ entering (2.1) ensures that the count starts at zero for *t*=1900, that is in the year before Nobel prizes were first awarded.

The parameters entering the sociophysical model (2.1) can be determined via a straightforward least square fit. For $\left(t_{\max},\tau_{\max}\right)$ one obtains (1909,5) for France, (1898,15) for Germany, (1995,5) for the UK and (1972,29) for the USA. The corresponding results for (*γ*,*α*) are (−0.34,135) for the USA, (0.98,−18.8) for the UK, (0.24,84) for Germany and (0.2,22.4) for France.

The set of parameters contains two particular cases. The negative *γ*<0 implies for the USA that a long-term productivity rate cannot be extracted, namely, that the boost ∼*α* completely dominates the US history of science Nobel prizes. For the UK one obtains that *α*<0, which indicates that the country experienced in the 1990s a phase of reduced (and not of increased) Nobel prize productivity.

**Figure 3.**
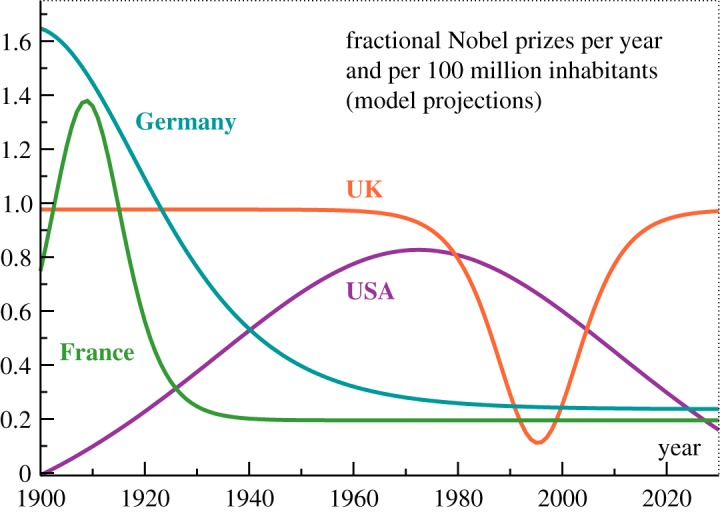
Historical Nobel productivity. The average number of fractional science Nobel prizes received per year and per 100 million inhabitants, as given by the derivative $\gamma +\alpha g(1-g)/\tau_{\max}$ of the respective analytic models. Compare equation (2.1) and figure 2.

The curves corresponding to (2.1), which have been included in figure 2, approximate the data to an astonishing degree, in particular for the USA. This observation suggests that the derivative of the analytic model with respect to time, namely $\gamma +\alpha g(1-g)/\tau_{\max}$, constitutes a reliable estimate for the evolving aggregate *per capita* Nobel prize productivity of a given country. The result is presented in figure 3. One finds that the four countries discussed here are surprisingly diverse with respect to how the efficiency of their scientific institutions evolved over the last century in terms of *per capita* Nobel productivity.

## 3. Discussion

Our results show that countries may vary drastically with respect to the historical development of their science productivity.

### 3.1. France

After a short boost in Nobel productivity that peaked in 1909, France produced Nobel laureates in physics, chemistry and medicine at a relatively constant rate of 0.2 per year and per 100 million inhabitants. The overall number of fractional medals received during 1901–2017 is 20. One needs to add that French scientific institutions excel furthermore in areas that do not affect the Nobel prize yield, in particular in mathematics. 13/12/6/1 Fields Medals were won, respectively, by the USA/France/UK/Germany. With a 2017 population of 65 million, France has by far the highest per *capita* success rate.

### 3.2. Germany

Germany’s science productivity peaked in 1898, which antedates the first Nobel prize by three years. The Nobel prize hence came somewhat too late for Germany, which would have received a substantially larger amount of medals if the first prize had been awarded not in 1901, but 20 years earlier. Today’s rate is 0.24 science Nobel prizes per year and per 100 million inhabitants.

German science funding is focusing these days more and more on cooperative funding programmes that are *per se* a response to the diminishing returns observed in many mature research fields [12]. It is yet to been seen whether the concurring progressive marginalization of individual research will have a detrimental impact, in the long run, on science productivity at the highest level in terms of Nobel medals.

### 3.3. UK

The UK received during most of the last century a whopping 0.98 science Nobel prizes per year and per 100 million inhabitants. This remarkable streak was interrupted temporarily in the mid-1990s, but we caution that the value for the depth of this depression found by the analytic model is to be taken only as an order of magnitude estimate. It will be interesting to see whether the 1990s depression in the number of UK science Nobel prizes was a one-time event or whether it bodes rougher times ahead.

### 3.4. USA

The entire US history of science Nobel prizes can be interpreted in terms of a single large productivity boost peaking in 1972. The extraction of a long-term basic productivity rate, if such a rate should be present, is pre-empted by the extraordinary long decay time of 29 years. A steady state with regard to scientific research has yet to be reached.

The average number of fractional science Nobel prizes the USA receives presently per year and per 100 million inhabitants is 0.34, a respectable value, which is however down by a factor of 2.4 relative to the peak value of 0.83 reached in 1972. What is striking, moreover, is the continuing downward trend. Our model predicts that the US *per capita* productivity rate will have fallen below that of Germany by 2025 and below that of France by 2028. It is hard to imagine, given the remarkable accuracy of the analytic model, a scenario for which the final bottoming out of the US *per capita* productivity of science Nobel laureates would not occur at very low levels.

The decline of the US *per capita* productivity of science Nobel laureates has been masked hitherto by the concurring increase of the US population, which equalled a factor of 1.6 between 1972 and 2017 (from 208 to 327 million). The continuously growing population size allowed the USA to increase the overall count of fractional science Nobel prizes in the same period from 52 to an impressive 126.

One may argue that *per capita* levels are destined to fall in a world in which the overall population grows at an unabated pace and in which more countries than ever fund scientific research. While undoubtedly true, this argument falls short to explain the large differences robustly observed when comparing France, Germany and the UK to the USA. Other factors must hence determine the observed decline of the US *per capita* productivity of Nobel laureates in the natural sciences.

National science funding policies can be clearly successful regardless of the prospect of acquiring Nobel prizes, in particular because Nobel prizes do not cover new fields like computer science, a typical US domain [13]. Is the ongoing decline of the US *per capita* physics, chemistry and medicine Nobel prize success then cause for alarm or nothing else than an indication of a paradigm shift, that is a refocusing of research priorities towards new and more rewarding areas?

## 4. Conclusion

We have shown that the timeline of physics, chemistry and medicine Nobel prizes provides a reliable database that allows to extract both a tractable model and to forecast future aggregate developments. We have pointed out in particular that the US *per capita* success rate has been declining ever since 1972 and that this terminal trend has been masked hitherto by the concurring growth of the population. The resulting prediction, namely that this downward trend will continue for the decade to come, allows to either corroborate or to invalidate the model proposed here. Future investigations may take into account the time lag that passes between a discovery and its honouring by a Nobel medal [14].

## Footnotes

1 The Nobel Committee. See http://www.nobelprize.org/.

2 United States Census Bureau. See http://www.census.gov/.

3 Office for National Statistics of the UK. See http://www.ons.gov.uk/.

4 Bundeszentrale für politische Bildung. See http://www.bpb.de/.

5 Institut national de la statistique et des études économiques. See https://www.insee.fr/.

## Data accessibility

This article has no additional data.
